# Development and validation of an ensemble artificial intelligence model for comprehensive imaging quality check to classify body parts and contrast enhancement

**DOI:** 10.1186/s12880-022-00815-4

**Published:** 2022-05-13

**Authors:** Seongwon Na, Yu Sub Sung, Yousun Ko, Youngbin Shin, Junghyun Lee, Jiyeon Ha, Su Jung Ham, Kyoungro Yoon, Kyung Won Kim

**Affiliations:** 1 Department of Computer Science and Engineering, Konkuk University, Seoul, Korea; 2 Clinical Research Center, Asan Medical Center, Seoul, Korea; 3 Department of Convergence Medicine, University of Ulsan College of Medicine, Seoul, Korea; 4 Biomedical Research Center, Asan Institute for Life Sciences, Asan Medical Center, Seoul, Korea; 5 Department of Medical Science, Asan Medical Institute of Convergence Science and Technology, Asan Medical Center, University of Ulsan College of Medicine, Seoul, Korea; 6 Department of Radiology, Hallym University College of Medicine, Kangdong Seong-Sim Hospital, Seoul, Korea; 7 Department of Radiology and Research Institute of Radiology, University of Ulsan College of Medicine, Asan Medical Center, 88 Olympic-ro 43-gil, Songpa-gu, Seoul, 05505 Republic of Korea; 8 Department of Smart ICT Convergence Engineering, Konkuk University, 120 Neungdong-ro, Gwangjin-Gu, Seoul, Republic of Korea

**Keywords:** Artificial intelligence, Computed tomography series, Deep learning, Transfer learning, Two-dimensional convert method, Image quality check

## Abstract

**Background:**

Despite the dramatic increase in the use of medical imaging in various therapeutic fields of clinical trials, the first step of image quality check (image QC), which aims to check whether images are uploaded appropriately according to the predefined rules, is still performed manually by image analysts, which requires a lot of manpower and time.

**Methods:**

In this retrospective study, 1669 computed tomography (CT) images with five specific anatomical locations were collected from Asan Medical Center and Kangdong Sacred Heart Hospital. To generate the ground truth, two radiologists reviewed the anatomical locations and presence of contrast enhancement using the collected data. The individual deep learning model is developed through InceptionResNetv2 and transfer learning, and we propose Image Quality Check-Net (Image QC-Net), an ensemble AI model that utilizes it. To evaluate their clinical effectiveness, the overall accuracy and time spent on image quality check of a conventional model and ImageQC-net were compared.

**Results:**

ImageQC-net body part classification showed excellent performance in both internal (precision, 100%; recall, 100% accuracy, 100%) and external verification sets (precision, 99.8%; recovery rate, 99.8%, accuracy, 99.8%). In addition, contrast enhancement classification performance achieved 100% precision, recall, and accuracy in the internal verification set and achieved (precision, 100%; recall, 100%; accuracy 100%) in the external dataset. In the case of clinical effects, the reduction of time by checking the quality of artificial intelligence (AI) support by analysts 1 and 2 (49.7% and 48.3%, respectively) was statistically significant (*p* < 0.001).

**Conclusions:**

Comprehensive AI techniques to identify body parts and contrast enhancement on CT images are highly accurate and can significantly reduce the time spent on image quality checks.

**Supplementary Information:**

The online version contains supplementary material available at 10.1186/s12880-022-00815-4.

## Background

Medical imaging has greatly advanced in various therapeutic fields, and computed tomography (CT) is used the most, especially in the field of oncology [1, 2]. In the field of clinical trial imaging, regulatory agencies seek robust evidence for imaging to emphasize the quality of image data [3]. In 2018, the US Food and Drug Administration issued a “Clinical Trial Imaging Endpoint Process Standard Guidance for Industry,” which provides current optimal procedures in using imaging in clinical trials, including image transfer, receive, archive, and quality check [4]. In multicenter clinical trials, numerous CT images are transferred from each site or hospital to a central server; thus, the image quality check must be performed by a central imaging team to reduce imaging defects or violations [5].

In multicenter clinical trials, the first step of image quality check is to check whether images are uploaded appropriately according to the predefined rules. For example, when the site clinical research coordinators (CRCs) are supposed to upload the contrast-enhanced abdomen-pelvic CT in a multi-center clinical trial, the image analyst should check whether the uploaded images are contrast-enhanced abdomen-pelvic CT or not. Thus, in this study, the first step of image quality check is hereafter referred to as image QC. 

Image QC was only performed manually by image analysts, and this requires human resources and time. Recently, an artificial intelligence (AI) technique has greatly advanced for imaging [6]. If an AI can aid in the automation of image QC, it will greatly help in reducing the resources for clinical trials. An image QC for the appropriateness of a sequence or slice thickness can be done by managing DICOM header files, whereas the appropriateness of body parts and contrast enhancement should be checked manually by image analysts.

AI techniques for image QC have been recently reported [7, 8]. However, these focused only on a part of image QC, which had low accuracy. Thus, we aimed to develop comprehensive AI techniques to classify body parts and contrast enhancement simultaneously and to validate the accuracy and clinical effectiveness of these techniques.

## Methods

This study was approved by the institutional review board of Asan Medical Center (AMC) and Kangdong Sacred Heart (KSH) Hospital. As this is a retrospective study, informed consent was not required. The results in this study were reported according to the methods and terms in published literature guidance on machine learning for medical applications [9].

### Data source and datasets

This is a retrospective study. From the picture archive and communication system of our institution (PetaVision), CT images of 1024 patients (464 women and 560 men; mean age, 60.4 ± 13.8 years) who underwent brain, neck, chest, abdomen, and abdominopelvic CT scans from May 1 to May 2 were obtained. An external validation set from 301 patients (113 women and 188 men; mean age, 65.4 ± 14.6) was obtained at the KSH hospital. The characteristics of the patients included in this study are summarized in Table 1. In some patients, multiple CT scans were obtained (e.g., chest CT and abdomen CT). From here, the unit of datasets was regarded as the number of CT scans.

**Table 1:** Summary of demographic variables for development, internal validation, and external validation

Variables	Development set (n = 923/1042)*		Internal validation set (n = 101/179)		External validation set (n = 301/448)	
*Demographics*						
Female (%, female:male)	46.3 (427:496)		36.6 (37:64)		37.5 (113:188)	
Age	60.3 ± 14.0		60.5 ± 12.1		65.4 ± 14.6	
Height (cm)	163.0 ± 9.1		163.8 ± 8.9		162.7 ± 9.8	
Weight (g)	62.4 ± 12.2		64.2 ± 13.8		62.1 ± 13.2	
BMI	23.5 ± 3.7		23.8 ± 3.8		23.4 ± 4.0	

Disease category	Patients	CT scan	Patients	CT scan	Patients	CT scan
Cancer	558	620	71	126	62	89
Benign tumor	66	70	8	8	2	2
Infection	62	87	5	19	46	97
Hemorrhage	11	11	1	1	33	41
Cirrhosis	14	17	3	6	21	49
Stone	28	36	0	0	4	6
Cyst	16	18	1	1	0	0
Transplant	19	22	1	1	0	0
Blunt trauma	2	2	0	0	33	50
Health check-up	147	159	11	17	100	114

^*^ Number of patients/CT scans

**Fig. 1:**
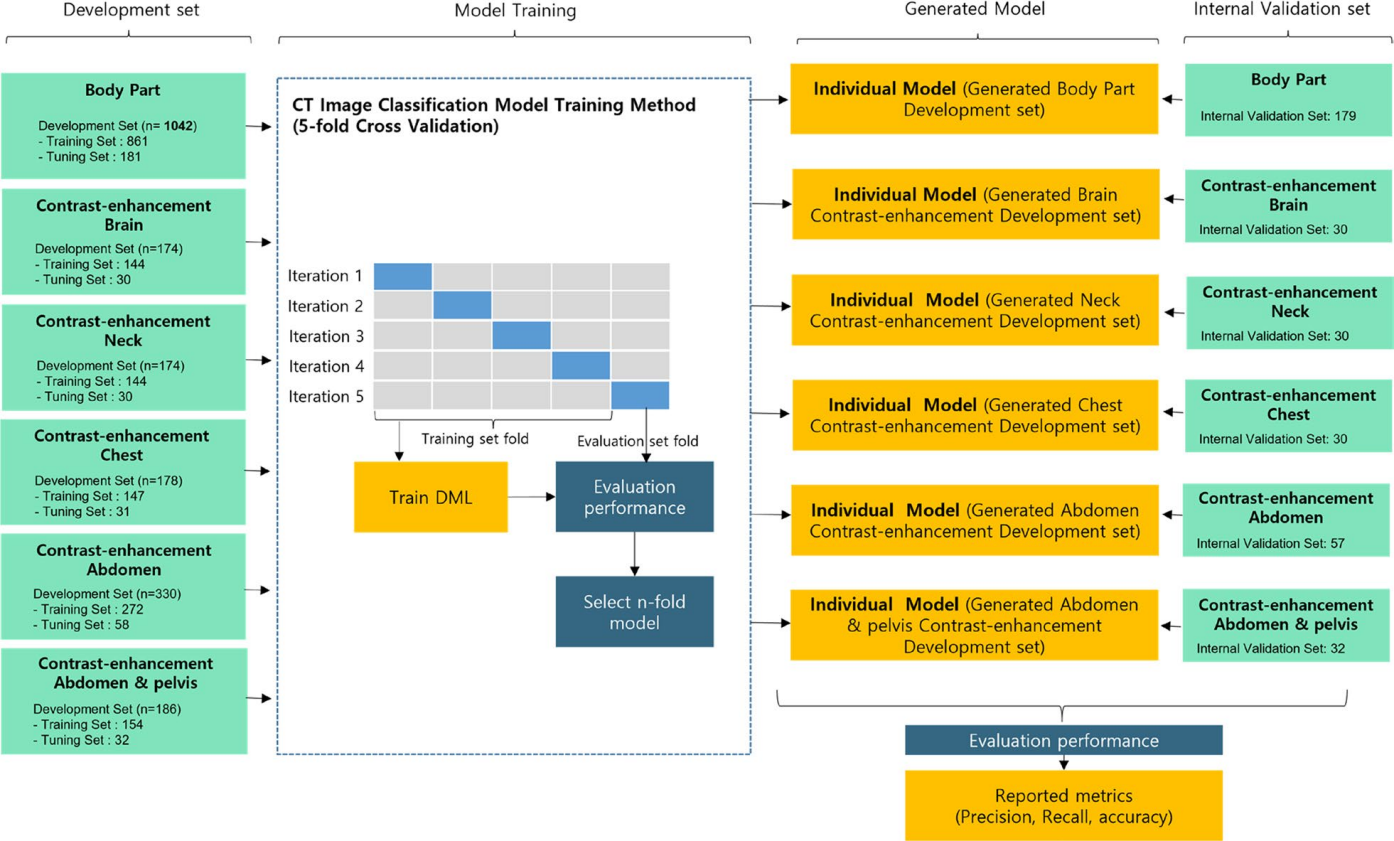
Details of deep learning model training and internal validation

The CT scans were randomly divided into a development set (n = 1042 scans) and an internal validation set (n = 179 scans). The detailed number of development sets and internal validation sets are presented in Fig. 1. The development dataset was split into a training set and tuning set at a ratio of 8:2, respectively. The training set was used for the development of a deep learning algorithm, whereas the tuning set was used to tune the hyperparameters of the model. The internal validation set (n = 179 scans) was used only for the independent test of developed models and not for training.

The external validation set (n = 448 scans) was prepared to compare the human experts from the deep learning model (DLM). Detailed information about the external validation set is summarized in Additional file 1: Supplementary Table 1. and Additional file 1: Supplementary Figure 1.

### Generation of the ground truth

The body part of the CT scans and presence of contrast enhancement were recorded by two board-certified radiologists (K.W.K for datasets obtained from AMC and J.Y.H. for datasets obtained from KSH). The two radiologists also analyzed the possible cause of misclassified cases by deep learning models (DLM).

### Design of ImageQC-net architecture

**Fig. 2:**
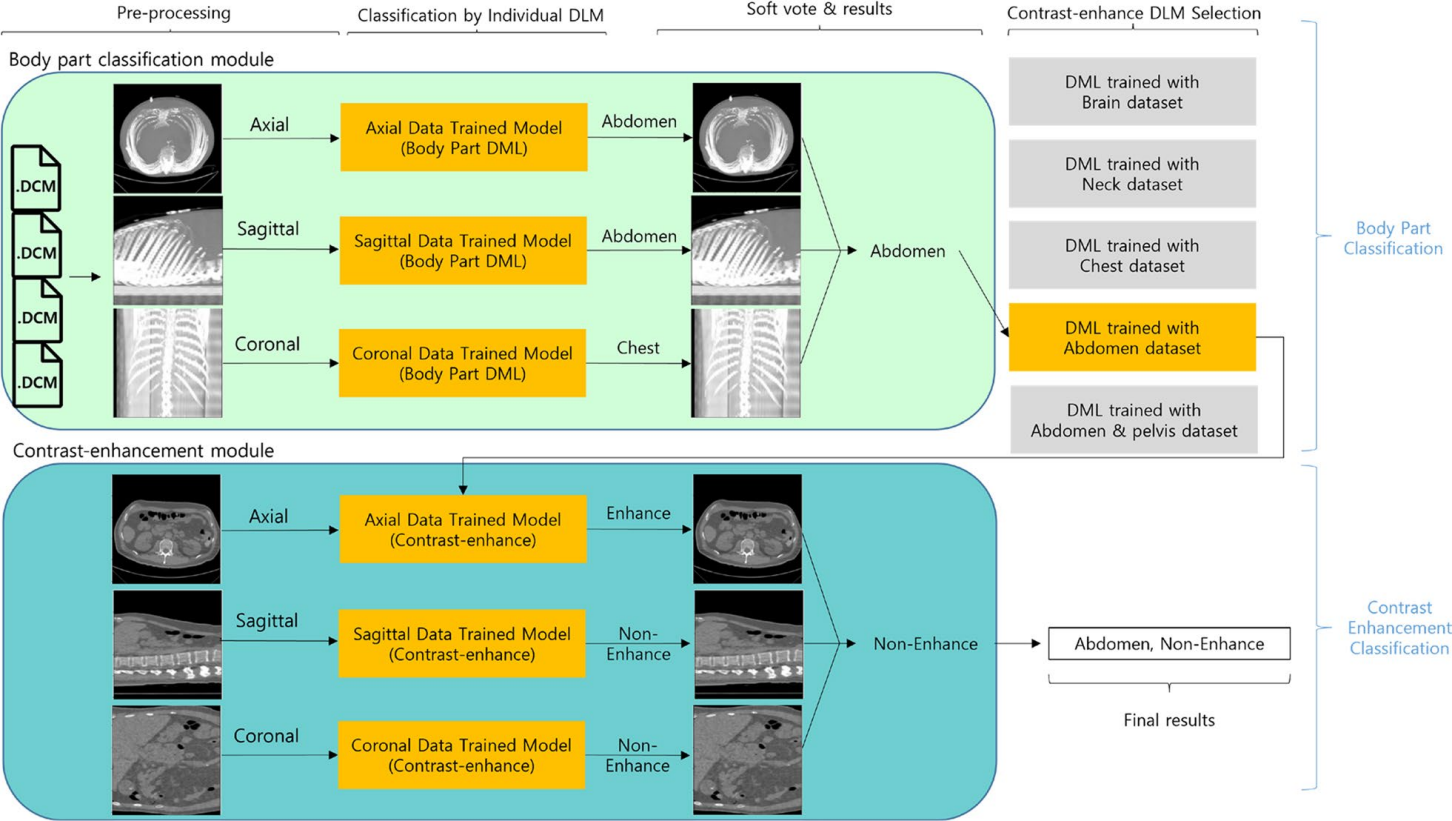
Overview of the ImageQC-net pipeline

The Fig. 2 illustrated the overall architecture of our ensemble AI model which classifies the body part and the use of contrast agents. Our ensemble AI model was named as ImageQC-net. Our ImageQC-net was composed of two separate modules: the body part classification module and the contrast-enhancement classification module. In each module, there were three processing steps: (1) Pre-processing to converted images from DICOM to PNG format in three perpendicular planes, (2) Individual DLM to yield prediction probability for classification in each plane, and (3) Ensemble AI model for final classification decision by using soft voting based on prediction probability of three different planes.

The ensemble AI model with soft voting based on DLM results of three different planes (axial, coronal, and sagittal images) was adopted because the information held by axial, coronal, and sagittal data is different and has information that cannot be seen in other planes. Thus, we proposed the ensemble AI model to combine individual model predictions to make a final prediction.

### Pre-processing

At first, preprocessing was executed to transform 3D image data with multiple slices into a 2D representative image using either maximum intensity projection (MIP), average intensity projection (AIP), or mid-plane.

For each CT scan, MIP images were reconstructed by selecting the voxels with maximum intensity that fall in the way of parallel rays traced from three perpendicular planes: axial (MIP_axial_), sagittal (MIP_sagittal_), and coronal (MIP_coronal_) plane. AIP images were reconstructed by averaging the voxels with maximum intensity that fall in the way of parallel rays traced from the three perpendicular planes: axial (AIP_axial_), sagittal (AIP_sagittal_), and coronal (AIP_coronal_) plane. Mid-plane images were selected from three planes—mid-axial, mid-sagittal, and mid-coronal plane [10].

**Fig. 3:**
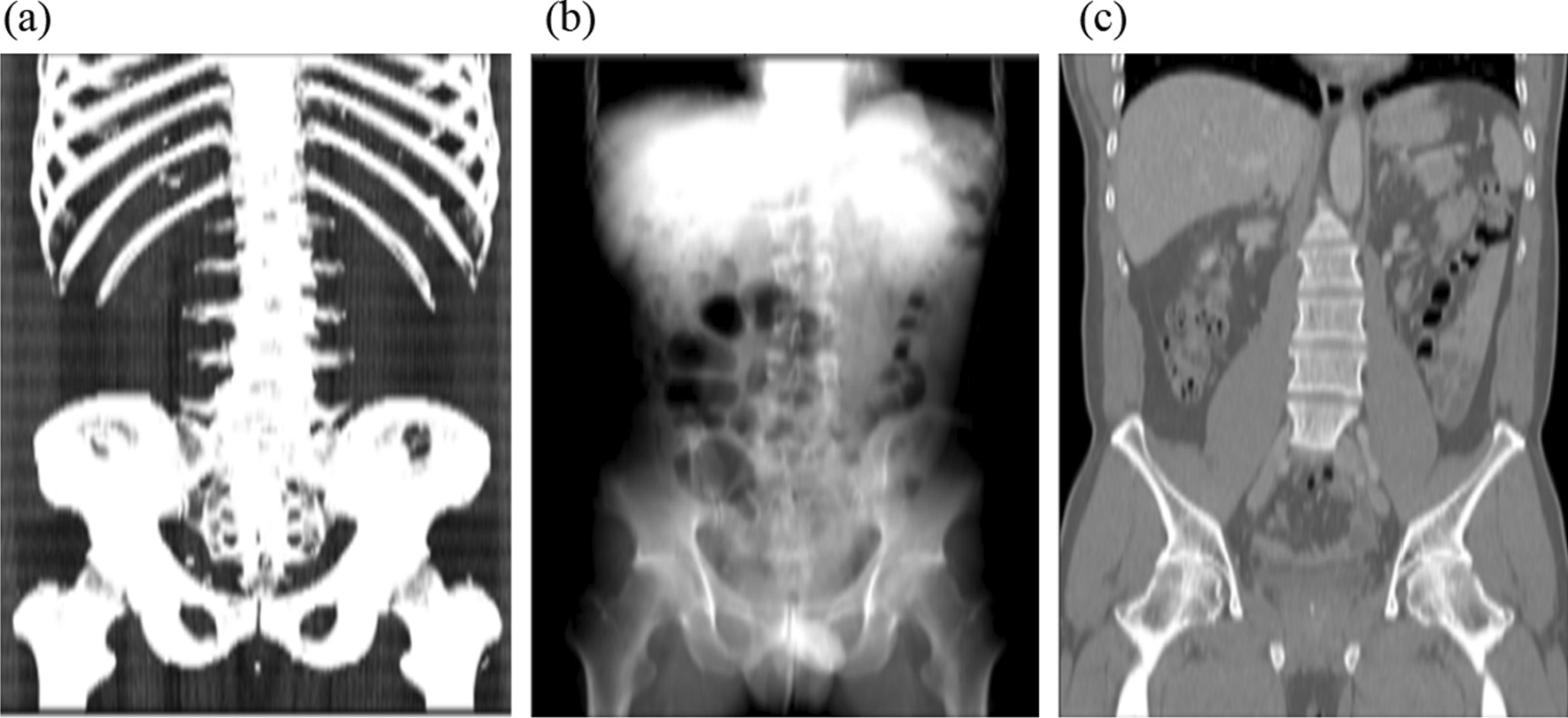
The image reconstruction method used in this study. The 3D CT image data with multiple slices are transformed into a 2D representative images using the maximum intensity projection in a coronal image (MIP_coronal_) (**a**), average intensity projection (AIP_coronal_) (**b**), and mid-coronal plane (**c**). In this example, we used a contrast-enhanced abdomen and pelvis CT

The DICOM images of the CT scans were converted into one-channel grayscale images in Portable Network Graphics (PNG) format as the standard image format. The window level and window widths were normalized to generate consistent grayscale information, irrespective of the scanner type and protocol [11, 12]. Set the window level to 50 and the window width to 1000 to produce consistent grayscale information regardless of scanner type and protocol. Subsequently the images were rescaled into a size of 512 × 512 pixels, and the pixel values were normalized to a range between zero and one. Figure 3 shows an example after the CT scan data is preprocessed.

### Development of individual DLM per plane

We developed several individual DLMs per plane for both the body part classification module and the contrast-enhancement classification module. For the body part classification module, DLMs were developed to classify images into one of the five body parts, including the brain, neck, chest, abdomen, abdomen & pelvis. For the contrast-enhancement classification module, DLMs were developed to classify the presence of contrast-enhancement. To measure the performance of each DLM, it was trained using the data sets obtained through the nine 2D conversion methods described in the preprocessing step.

All individual DLMs were developed by transfer learning with InceptionResNetV2 as the backbone. Inception architecture is known to achieve excellent performance at relatively low computational costs, and we chose it as a backbone because the combination with residual connections significantly accelerates the training of inception network [13]. Transfer learning is a widely used method in the field of computer vision because it can create accurate models in a short time. DLMs are set to the same hyperparameters as shown in Additional file 1: Supplementary Table 2.

The layers of DLM are described in Additional file 1: Supplementary Figure 2. In the input layer, a CT series was converted into a 2D image using the preprocessing method described above. After the preprocessing stage, in the input layer, we arranged three channels (299 × 299 × 3) by copying the one-channel normalized image. The three channel images were fed into the pre-trained model layers. The InceptionResnetv2 model was adopted as pretrained model layers, which performed well in both learning speed and accuracy. The learning strategy was to update the weight of all layers of the backbone model, and these choices were based on the results of repeated experiments. The output layer was composed of a flatten layer and SoftMax layer. The number of classes was adjusted for each network (five classes for body part classification networks and two classes for contrast enhancement use detection networks).

In the development set, fivefold cross-validation was performed to check the generalization ability of the model. The development dataset (n = 1042 scans) was partitioned into five equal subgroups in a stratified manner. Of the five subgroups, a single subgroup was retained as the test data to evaluate the model, and the remaining four subgroups were used as the training data. The process was repeated five times, where each of the five subgroups was used exactly once as the test data. In addition, to reduce overfitting on the model, two distinct forms of data augmentation were employed in the training set—image rotation and flip. Using data augmentation, 8336 CT scans were generated from the 1042 CT scans. In the data augmentation method for rotation, the angles of rotation were randomly selected between − 20° and 20°. Horizontal and vertical flips were performed on all CT scans in the training set.

### Development of ensemble AI model

Ensemble AI model for final classification decision was developed by using soft voting based on prediction probabilities of selected individual DLMs from three different planes.

Equation (1) selects the highest class by summing the probabilities predicted by the individual DLM for each class. Here *y* represents the final prediction, *i* represents the number of classes, and *j* represents DLM. In the body part classification module, *i* is set to 5 and in the contrast-enhancement classification module, it is set to 2. The *j* value is set equally to 3 in both modules of ImageQC-net.

1 $$y = \mathop {\arg \max }\limits_{i} \mathop \sum \limits_{j = 1}^{m} p_{ij}$$

### Performance evaluation and statistical evaluation

In the internal and external validation sets, the performances of individual DLMs per plane and the ensemble AI model (i.e., ImageQC-net) in classifying the body parts and in classifying the presence of contrast-enhancement were evaluated. To evaluate the performance accuracy, precision, recall, and F1-score were used.

The accuracy, precision, and recall were defined as:

- $$Accuracy = \frac{{{\text{TP}} + {\text{FP}}}}{{{\text{TP}} + {\text{TN}} + {\text{FN}} + {\text{FP}}}}$$
- $$Precision = \frac{TP}{{TP + FP}}$$
- $$Recall = \frac{TP}{{TP + FN}}$$
- $${\text{F}}1{\text{-score}} = 2 \times ({\text{precision}} \times {\text{recall}})/({\text{precision}} + {\text{recall}})$$

### Clinical effectiveness evaluation

To evaluate the clinical effectiveness of ImageQC-net in classifying body parts and in identifying contrast enhancement, the overall accuracy and time spent on image QC for either conventional manual methods or ImageQC-net were compared. Two image analysts with different experience levels for image QC in clinical trials (Analyst 1: S.J.H., 9 years of experience; Analyst 2: S.Y.L., 1 month of experience) independently checked the body parts and presence of contrast enhancement in two different sessions with a 2-week interval. The first session was for conventional manual QC and image analysts were required to check, the body part and contrast-enhancement based on raw CT images. The second session was for AI-aided quality check and image analysts were required to check the body part and contrast-enhancement based on the AI label and summary images generated by the ImageQC-net. If necessary, image analysts reviewed the raw CT images.

## Results

### Performances of individual DLMs for body part classification

In the internal validation set, the individual DLMs showed excellent performance in all preprocessing methods and planes (accuracy, 100%), as presented in Table 2. In the external validation set, the DLMs of MIP_sagittal_ (accuracy, 99.33%), MIP_axial_ (accuracy, 98.66%), and MIP_coronal_ (accuracy, 98.66%) were selected for the ensemble algorithm for body part classification, as the DLMs of MIP preprocessing method achieved high performances in each plane compared to those of other preprocessing methods. The cross-validation performances of individual DLMs based on various preprocessing methods for body part classification are presented in Additional file 1: Supplementary Table 3.

**Table 2:** Performance of individual DLMs for body part classification: comparison among the algorithm models based on preprocessing methods and planes

Pre-processing Method	Internal validation set				External validation set			
	Precision (%)	Recall (%)	Accuracy (%)	F1-score (%)	Precision (%)	Recall (%)	Accuracy (%)	F1-score (%)
*AIP*								
Axial	100	100	100	100	94.99	94.15	94.86	94.6
Sagittal	100	100	100	100	97.52	96.85	97.09	97.2
Coronal	100	100	100	100	95.24	91.73	93.75	93.45
*MIP*								
Axial	100	100	100	100	98.85	98.33	98.66^a^	98.6
Sagittal	100	100	100	100	99.33	99.33	99.33^ab^	99.3
Coronal	100	100	100	100	98.85	98.33	98.66^a^	98.6
*Mid-plane*								
Axial	100	100	100	100	96.24	96.51	96.2	96.4
Sagittal	100	100	100	100	97.7	96.32	97.32	97
Coronal	100	100	100	100	96.24	96.51	96.2	96.3

^a^The DLMs with the highest performance in each plane are selected to apply for ensemble AI model

^b^The DLM with the highest performance from all pre-processing methods is selected as the best performing individual DLM

**Fig. 4:**
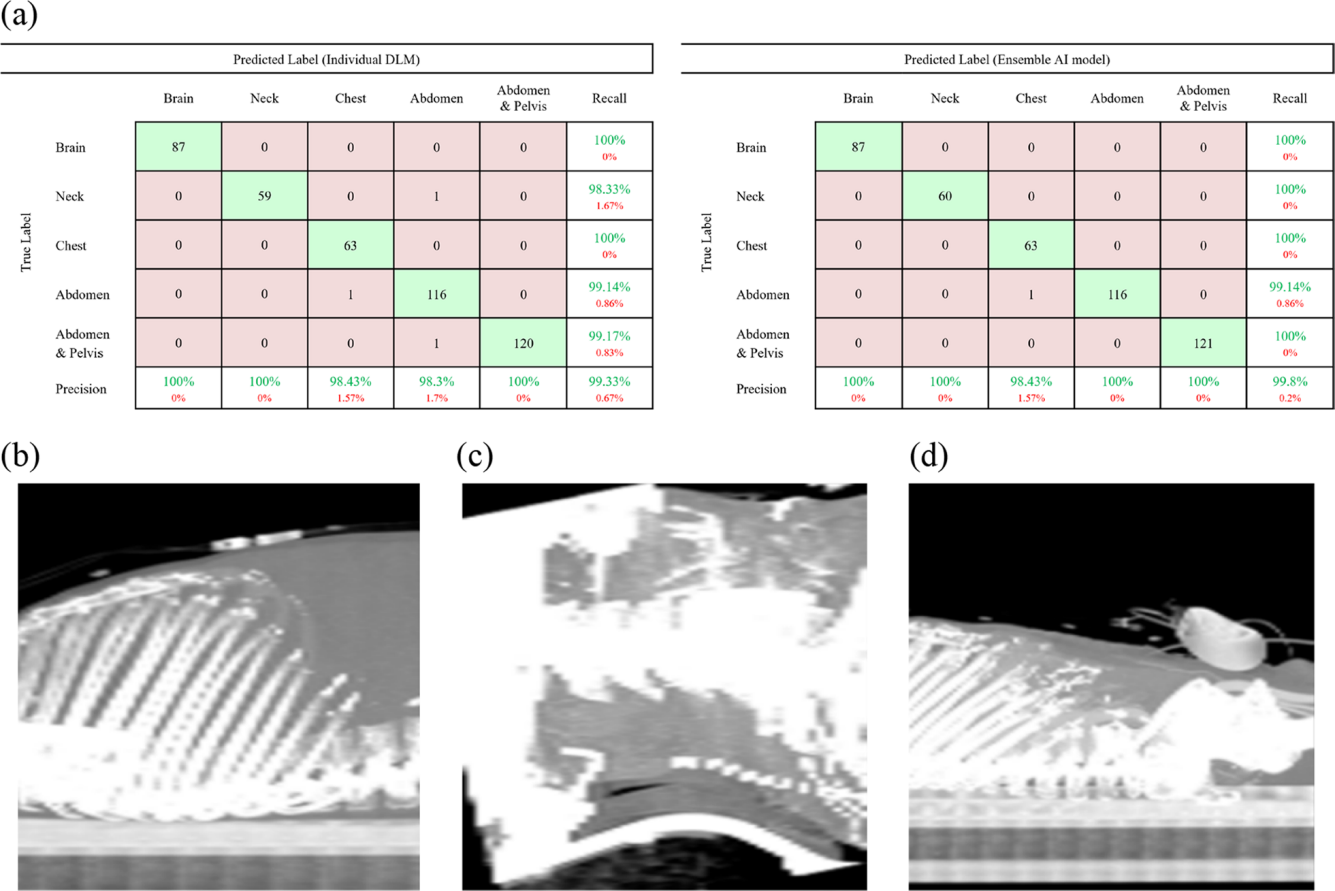
Comparison between the individual DLM and the ensemble AI model for body art classification based on the external dataset. **a** Confusion matrices of the best performing individual DLM and the ensemble AI model. **b** Abdominal CT misclassified as chest CT in both the best performing individual DLM and the ensemble AI model. **c** Neck CT misclassified as abdominal CT from the best performing DLM. **d** Abdominopelvic CT misclassified as abdominal CT from the best performing DLM

The confusion matrix of the best performing DLM (MIP_sagittal_) validated in the external validation set is presented in Fig. 4. There were three cases with discrepancies between AI-predicted results and ground truth results. These discrepancies were primarily attributed to the resemblance of MIP_sagittal_ images between abdominal CT and chest CT scan in a patient with diaphragm elevation (b), between neck CT and abdominal CT scan (case c), and between abdominal CT and abdominopelvic CT scan (d).

### Performances of ensemble AI model for body part classification

The performance of ensemble AI model for body part classification showed 100% precision, 100% recall, and 100% accuracy in the internal validation set, and 99.8% precision, 99.8% recall, and 99.8% accuracy in the external validation set. Among the 448 CT scan in the external validation set, only one CT showed discrepancy between AI result (classified as chest CT) and ground truth value (abdominal CT), as illustrated in Fig. 4b.

### Performance of individual DLMs for contrast-enhancement classification

The cross-validation results of individual DLMs in the tuning set are provided in Additional file 1: Supplementary Table 4. In the tuning set, the DLM algorithms based on mid-plane showed an excellent performance in all body parts (accuracy, 94–100%) compared with those based on MIP or AIP (accuracy, < 90%). Based on these cross-validation results, the candidate models for ImageQC-net were chosen from each preprocessing method. In each body part, the performance of DLM algorithms based on various preprocessing methods for contrast-enhancement classification is summarized in Table 3. When we compared the performance of DLM algorithms based on mid-plane with various planes, the performance differed in each body part, as shown in Table 3.

**Table 3:** Performance of individual DLMs for contrast-enhancement classification: comparison among the algorithm models in each body part

Body part	Preprocessing	Internal validation set				External validation set			
		Precision (%)	Recall (%)	Accuracy (%)	F1-score (%)	Precision (%)	Recall (%)	Accuracy (%)	F1-score (%)
Brain	Mid-axial	100	100	100	100	100	100	100	100
Neck		100	100	100	100	98.38	98.33	98.33	98.3
Chest		100	100	100	100	98.38	98.48	98.41	98.4
Abdomen		100	100	100	100	100	100	100	100
Abdomen and pelvis		100	100	100	100	97.78	97.87	98.34	97.9
Brain	Mid-sagittal	100	100	100	100	99.13	98.33	98.85	98.7
Neck		100	100	100	100	84.09	76.66	76.66	80
Chest		100	100	100	100	100	100	100	100
Abdomen		100	100	100	100	100	100	100	100
Abdomen and pelvis		100	100	100	100	100	100	100	100
Brain	Mid-coronal	100	100	100	100	99.13	98.33	98.85	98.7
Neck		100	100	100	100	91.17	91.66	91.66	91.4
Chest		100	100	100	100	100	100	100	100
Abdomen		100	100	100	100	100	100	100	100
Abdomen and pelvis		100	100	100	100	100	100	100	100

In the external validation set, the plane to obtain highest performance differed in each body part, as follows: mid-axial plane for brain CT (100%), mid-axial plane for neck CT (accuracy, 98.33%), mid-coronal plane for chest CT (100%), mid-coronal plane for abdominal CT (100%), and mid-coronal plane for abdominopelvic CT (100%). These results are primarily attributed to the anatomic characteristics of each body part. In the brain and neck CT, strongly enhancing organs, such as vessels, are demonstrated in the axial image. The coronal and sagittal neck CT images primarily show bones and muscles. In the chest, abdominal, and abdominopelvic CT, strongly enhancing organs, such as the heart, liver, and aorta, are best demonstrated in the mid-coronal plane images.

### Performances of ensemble AI model for contrast-enhancement classification

**Fig. 5:**
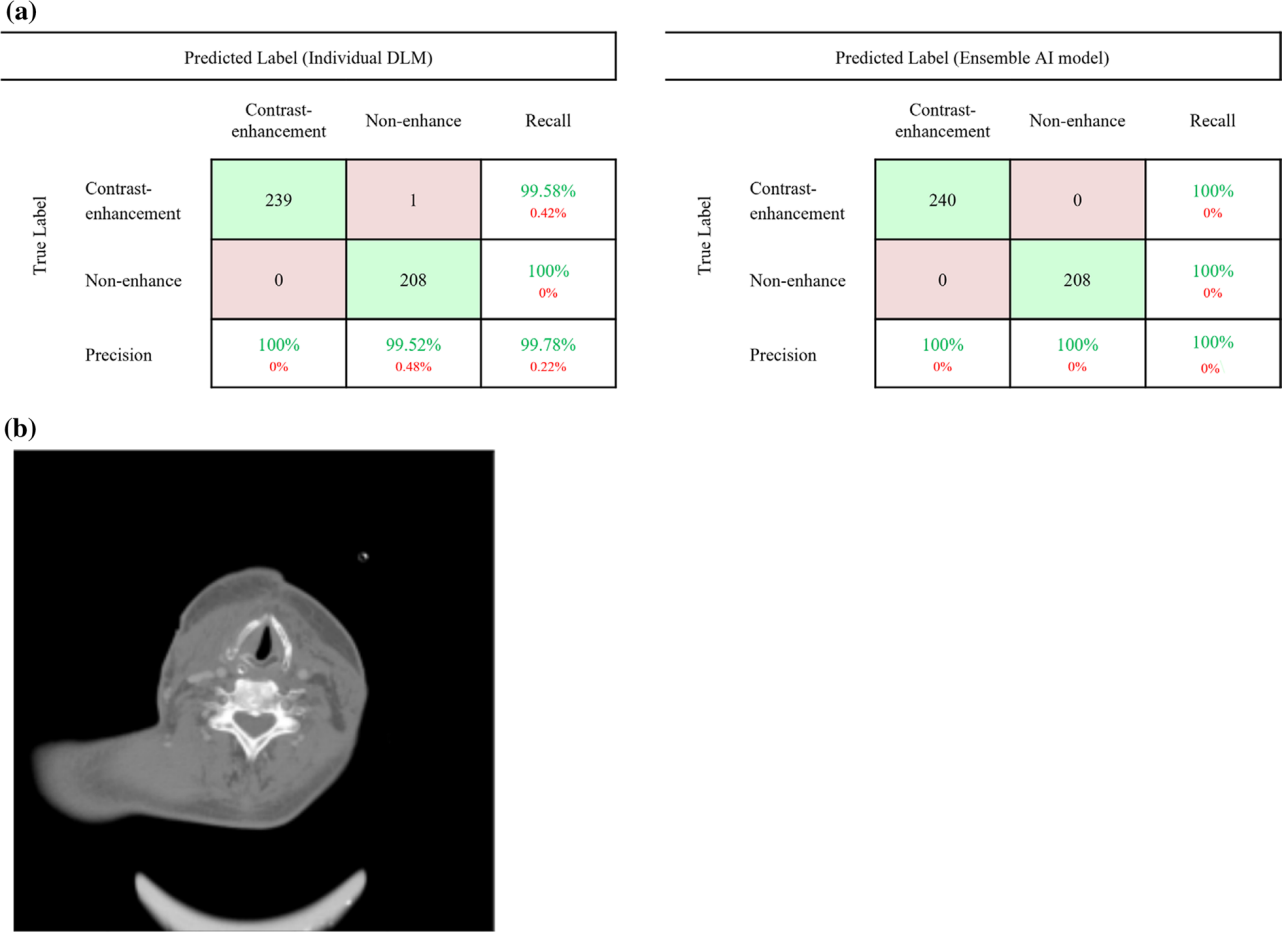
Comparison between the individual DLM and the ensemble AI model for contrast-enhancement classification based on the external dataset. **a** Confusion matrices of the best performing individual DLM and the ensemble AI model. **b** Misclassified case in the individual DLM. In the neck mid-plane CT image, the neck vessels are small; thus, the DLM algorithm may not identify contrast enhancement

The performance of ensemble AI model for contrast-enhancement classification showed 100% precision, 100% recall, and 100% accuracy in the internal and external validation sets. As shown in (b) of Fig. 5, cases misclassified in individual DLM was classified corrected in the ensemble AI model.

### Comparison of overall performance for body part and contrast-enhancement classifications

**Fig. 6:**
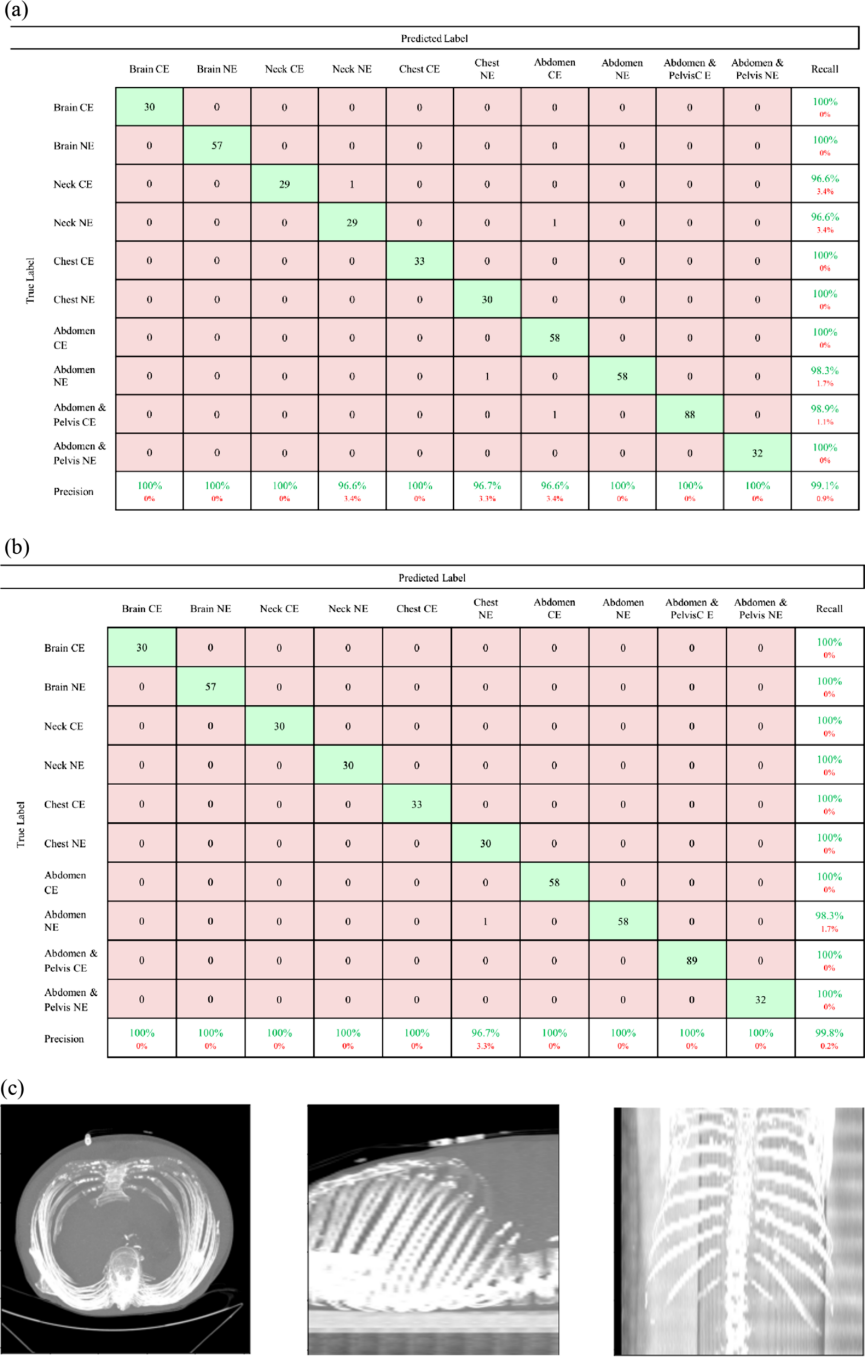
overall DLM and ensemble AI model classification performance for the external dataset. **a** Overall DLM classification performance for the external dataset. **b** Overall Ensemble AI model classification performance for the external dataset. **c** Ensemble AI model external dataset misclassified case

Figure 6 shows the overall performance of the best performing individual DLM and the ensemble AI model, ImageQC-net, in the external verification set. The overall accuracy of best performing individual DLM for proper classification of both body parts and contrast enhancement was 99.1%, as it misclassified 4 CT scans out of 448 CT scans. In contrast, the ImageQC-net based on ensemble AI models, demonstrated 99.8% overall accuracy by misclassifying only one CT scan out of 448 CT scans.

### Clinical effectiveness

**Fig. 7:**
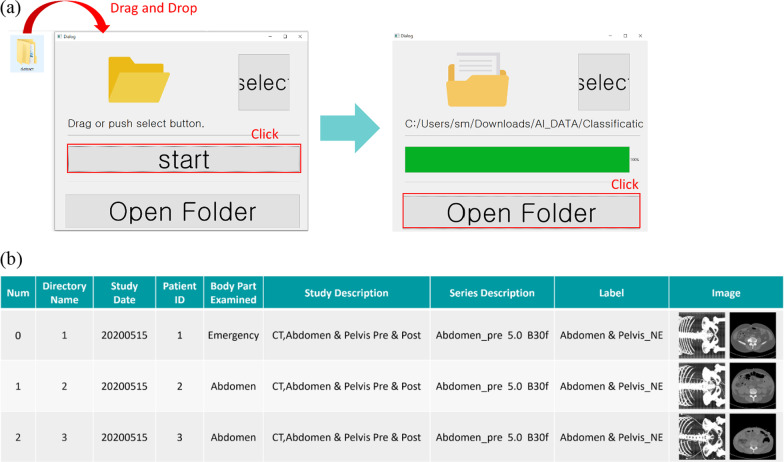
ImageQC-net GUI software. **a** ImageQC-net GUI software. **b** Results available to users

The ImageQC-net was published into a graphic user interface software package (Fig. 7). In the first session (conventional manual quality check), the time spent by analysts on checking the body parts and the presence of contrast enhancement in the external validation set (448 CT scans) was 149 min for analyst 1 and 205 min for analyst 2. In the second session (ImageQC-net aided quality check), the mean time spent was 74 min for analyst 1 and 99 min for analyst 2. The actual time spent by the ImageQC-net for the overall classification was 688 s for 448 CT scans (equivalent to 1.51 s/scan) using GPU RTX Titan. The time reduction by AI-aided quality check in both analyst 1 and 2 (49.7% and 48.3% decrease, respectively) was statistically significant (t-test, *p* < 0.001 for both analysts). The accuracy of the two image analysts was 100% in all the sessions. The one misclassified case by the ImageQC-net was corrected by the analysts in the second session.

## Discussion

This study demonstrated that the ensemble AI model, ImageQC-net, to check the body parts and contrast enhancement in the CT images was highly accurate (99.8%) and could greatly reduce the time spent for image QC (48.3–49.7% decrease). Our AI technique, named ImageQC-net, was designed to provide image QC results with two thumbnails of mid-plane image and MIP image so that image analysts can quickly identify the body parts and contrast enhancement of the CT images. More specifically, image analysis can skip scrolling down the CT images to check the scan coverage if ImageQC-net is used.

Indeed, the ImageQC-net was incorporated as a central imaging core lab in our clinical trial image management system (Asan Image Metrics, www.aim-aicro.com) to assist our image analysts [14]. Based on our real-world experience, the ImageQC-net is also greatly helpful for the image QC by image analysts, who should check the quality of the received CT images, and for image senders, who should transfer CT images to the central imaging core lab. For example, when a site or hospital is supposed to transfer the neck, chest, and abdominopelvic CT scans, but a clinical research coordinate (CRC) or research nurse missed to transfer the neck CT scans, a CRC may transfer the non-contrast abdominal CT scan only when contrast-enhanced abdominopelvic CT scan is required for a clinical trial. In these situations, the image senders, such as the CRC, can quickly check the CT images immediately before image transfer using the ImageQC-net. Our future research topic is to further evaluate the benefit of our ImageQC-net in the real-world setting.

The accuracy of ImageQC-net was very high (99.8%, 447/448), which exceed the accuracy of best performing DLM models (99.1%, 444/448).

In the best performing DLM model, the primary reason of misclassification might be attributable to a selected plane. For example, it adopted the best model for body part classification based on MIP_sagittal_ images, but the MIP_sagittal_ images between chest CT and abdomen CT or between abdominal CT and abdominopelvic CT may be similar. Moreover, the best performing DLM adopted the best model for contrast-enhancement classifications based on mid-axial plane images for brain and neck CT scans and mid-coronal plane images for chest, abdominal, and abdominopelvic CT scans. More specifically, a differentiation between chest and abdomen CT scans can be overlapped with patients’ anatomy. For example, in patients with kyphosis or diaphragm elevation, the MIP_sagittal_ image of both chest CT and abdomen CT scan would show most of the bony thorax, which may cause misclassification. Between abdominal and abdominopelvic CT scans, the coverage of the pelvic bone might be tricky even for expert humans. Interestingly, the neck and pelvic CT scans may show similar bone structures; the scapula and cervical spine bones may resemble the iliac bone and lumbar spine bones in MIP images. Mid-plane images may not contain strongly enhancing organs, which hamper the accuracy of contrast-enhancement classification. For example, in the neck, only the thyroid glands are the enhancing solid organs, but these may not be included in mid-plane images. In neck mid-axial plane images, only vessels can be the enhancing structures, but these are small, which may hamper the classification accuracy of the AI algorithm.

To overcome these drawbacks of best performing individual DLM, an ensemble approach to combine various models from various planes increase the classification accuracy, as proven in our study.

So far, only a few studies have developed an AI algorithm for image QC. Philbrick et al. reported that they developed a classifier model trained to predict contrast-enhancement phases from CT images based on a convolutional neural network (CNN), which can generate gradient-weighted class activation maps (Grad-CAMs) for whole-slice abdominal CT data [7]. The classifier was developed only for multiphasic abdominal CT images, which may limit its application in real-world practice for comprehensive image QC. Sugimori et al. reported that a classifier was developed to classify the body parts and contrast enhancement using the CNN architecture of AlexNet and GoogLeNet. However, the accuracy of these models was not high (72.1–86.2%), hampering its application in real-world practice [8].

To incorporate the AI-aided image QC solution in real-world practice, our development strategy was to achieve a high accuracy while balancing the computing power to run the AI algorithm on a personal computer. Thus, a 2D-based AI algorithm was chosen rather than 3D or whole-slice data-based algorithm. Then, the optimal representative 2D image plane for the AI algorithm was determined through extensive repeated experiments, and ensemble AI model was found to be the best for both body part classification and contrast-enhancement classification.

In our study, transfer learning was adopted [11]. As a pretrained model, InceptionResnetV2 [13] was used, which was selected through repeated experiments [15]. Among the pretrained models, VGG16 took a long time to learn many parameters and was less accurate (< 90%); the Inception line had fewer parameters, so the learning time was fast, but InceptionResnetV2 achieved the best accuracy.

The ImageQC-net does not require a high computing power. A conventional personal computer or laptop with a CPU only can be used. For example, the clinical effectiveness session to measure the time spent for image QC was performed using a personal computer with Intel i3-10100 CPU 3.6 GHz (without GPU) and a laptop with Intel i7-6500 CPU 2.5 GHz (without GPU).

### Limitations

First, the ImageQC-net was tested in a relatively small number of CT scans (179 CT scans for internal validation and 448 CT scans for external validation). The external validation set was acquired from only one external institution. Thus, a large-scale validation study might be required. Second, the ImageQC-net was trained based on brain, neck, chest, abdominal, and abdominopelvic CT scans, excluding the extremity or spine CT scans. Thus, it is necessary to update the ImageQC-net to check all kinds of CT scans. Third, we developed AI algorithm for only the first step of image QC, which is to check whether images are uploaded appropriately according to the predefined rules. We did not develop to find out presence of image artifacts or low spatial resolution, which is our future research topic.

## Conclusions

An integrated ensemble AI model to classify body parts and contrast enhancement simultaneously is feasible. Our ImageQC-net is currently used in routine clinical practice and is very helpful to reduce the time spent for image QC for both image senders and receivers.

## Supplementary Information

**Additional file 1.** **Supplementary Table 1.** Number of CT scans for development, internal validation, and external validation. **Supplementary Table 2.** Hyperparameters for DLM Training. **Supplementary Table 3.** Average and standard deviation of performance measurement (%) of individual DLMs for body part classification in 5-fold cross-validation. **Supplementary Table 4.** Average and standard deviation of performance measurement of contrast-enhancement classification models (%). **Supplementary Figure 1.** Detailed information of external validation set. **Supplementary Figure 2.** Training scheme of individual DLM.

## Data Availability

The datasets generated and analyzed during the current study are not publicly available due to the security of data but are available from the corresponding author upon reasonable request.

## Abbreviations

2D: Two dimensions
3D: Three dimensions
AI: Artificial intelligence
AIP: Average intensity projection
AMC: Asan Medical Center
CNN: Convolutional neural network
CT: Computed tomography
DICOM: Digital imaging and communications in medicine
DLM: Deep learning model
FN: False negative
FP: False positive
GPU: Graphics processing unit
GUI: Graphical user interface
Image QC: Image quality check
ImageQC-net: Image quality check network
KSH: Kangdong Sacred Heart Hospital
MIP: Maximum intensity projection
TN: True negative
TP: True positive

## References

[1] Lee AJ, Kim KW, Shin Y, Lee J, Park HJ, Cho YC, Ko Y, Sung YS, Yoon BS (2021). CDISC-compliant clinical trial imaging management system with automatic verification and data transformation: focusing on tumor response assessment data in clinical trials. J Biomed Inform.

[2] Park HY, Kim KW, Yoon MA, Lee MH, Chae EJ, Lee JH, Chung HW, Yoon DH (2020). Role of whole-body MRI for treatment response assessment in multiple myeloma: comparison between clinical response and imaging response. Cancer Imaging.

[3] Yankeelov TE, Mankoff DA, Schwartz LH, Lieberman FS, Buatti JM, Mountz JM, Erickson BJ, Fennessy FM, Huang W, Kalpathy-Cramer J (2016). Quantitative Imaging in Cancer Clinical Trials. Clin Cancer Res.

[4] US, Food, and, Drug, Administration: clinical trial imaging endpoint process standards guidance for industry; 2018.

[5] Gierada DS, Garg K, Nath H, Strollo DC, Fagerstrom RM, Ford MB (2009). CT quality assurance in the lung screening study component of the National Lung Screening Trial: implications for multicenter imaging trials. AJR Am J Roentgenol.

[6] Kim DW, Jang HY, Kim KW, Shin Y, Park SH (2019). Design characteristics of studies reporting the performance of artificial intelligence algorithms for diagnostic analysis of medical images: results from recently published papers. Korean J Radiol.

[7] Philbrick KA, Yoshida K, Inoue D, Akkus Z, Kline TL, Weston AD, Korfiatis P, Takahashi N, Erickson BJ (2018). What does deep learning see? Insights from a classifier trained to predict contrast enhancement phase from CT images. Am J Roentgenol.

[8] Sugimori H (2018). Classification of computed tomography images in different slice positions using deep learning. J Healthc Eng.

[9] Liu Y, Chen PC, Krause J, Peng L (2019). How to read articles that use machine learning: users' guides to the medical literature. JAMA.

[10] Sato Y, Shiraga N, Nakajima S, Tamura S, Kikinis R (1998). Local maximum intensity projection (LMIP: a new rendering method for vascular visualization. J Comput Assist Tomogr.

[11] Park HJ, Shin Y, Park J, Kim H, Lee IS, Seo D-W, Huh J, Lee TY, Park T, Lee J (2020). Development and validation of a deep learning system for segmentation of abdominal muscle and fat on computed tomography. Korean J Radiol.

[12] Schneider U, Pedroni E, Lomax A (1996). The calibration of CT Hounsfield units for radiotherapy treatment planning. Phys Med Biol.

[13] Szegedy C, Ioffe S, Vanhoucke V, Alemi A. Inception-v4, inception-resnet and the impact of residual connections on learning. In: Proceedings of the AAAI conference on artificial intelligence; 2017.

[14] Shin Y, Kim KW, Lee AJ, Sung YS, Ahn S, Koo JH, Choi CG, Ko Y, Kim HS, Park SH (2019). A good practice-compliant clinical trial imaging management system for multicenter clinical trials: development and validation study. JMIR Med Inform.

[15] Kornblith S, Shlens J, Le QV. Do better imagenet models transfer better? In: Proceedings of the IEEE/CVF conference on computer vision and pattern recognition; 2019. p. 2661–71.

