# Supplementary material for: Development and validation of an ensemble artificial intelligence model for comprehensive imaging quality check to classify body parts and contrast enhancement
Source: BMC Med Imaging. 2022 May 13;22:87. doi: 10.1186/s12880-022-00815-4 (PMC9107169; doi:10.1186/s12880-022-00815-4)
Supplement: Supplementary file 1 — Additional file 1. Supplementary Table 1. Number of CT scans for development, internal validation, and external validation. Supplementary Table 2. Hyperparameters for DLM Training. Supplementary Table 3. Average and standard deviation of performance measurement (%) of individual DLMs for body part classification in 5-fold cross-validation. Supplementary Table 4. Average and standard deviation of performance measurement of contrast-enhancement classification models (%). Supplementary Figure 1. Detailed information of external validation set. Supplementary Figure 2. Training scheme of individual DLM. [file 12880_2022_815_MOESM1_ESM.docx]

| **Supplementary Table 1. Number of CT scans for development, internal validation, and external validation.** | | | | | | | | | | | |
| --- | --- | --- | --- | --- | --- | --- | --- | --- | --- | --- | --- |
| **Datasets for contrast-enhancement classification** | | Brain | | Neck | | Chest | | Abdomen | | Abdomen & Pelvis | |
|  |  | CE | NE | CE | NE | CE | NE | CE | NE | CE | NE |
| Development set | —Training | 72 | 72 | 72 | 72 | 74 | 73 | 196 | 76 | 77 | 77 |
|  | —Tuning | 15 | 15 | 15 | 15 | 16 | 15 | 42 | 16 | 16 | 16 |
| Internal validation set | | 15 | 15 | 15 | 15 | 15 | 15 | 41 | 16 | 16 | 16 |
| External validation set | | 30 | 57 | 30 | 30 | 33 | 30 | 58 | 59 | 89 | 32 |

| **Supplementary Table 2. Hyperparameters for DLM Training.** | |
| --- | --- |
| Training epochs | 200 |
| Mini-batch size | 6 |
| Solver type | Stochastic Gradient Descent (SGD) |
| Base learning rate | 0.001 |
| Momentum | 0.9 |
| Early Stopping | 5 |
| Loss Function | CrossEntropyLoss |
| Weight Initialization | ImageNet |
| Trainable | All Layer |
|  |  |

| **Supplementary Table 3. Average and standard deviation of performance measurement (%) of individual DLMs for body part classification in 5-fold cross-validation.** | | | | |
| --- | --- | --- | --- | --- |
| Preprocessing Method | | Cross-validation performances in tuning set* | | |
|  |  | Precision | Recall | Accuracy |
| **AIP** | Axial | 100 ± 0 | 100 ± 0 | 100±0 |
|  | Sagittal | 99.89 ± 0.1 | 99.82 ± 0.2 | 99.85±0.2 |
|  | Coronal | 99.35 ± 0.4 | 99.54 ± 0.3 | 99.41±0.4 |
| **MIP** | Axial | 100 ± 0 | 100 ± 0 | 100 ± 0 |
|  | Sagittal | 99.88 ± 0.2 | 99.82 ± 0.2 | 99.85 ± 0.2 |
|  | Coronal | 100 ± 0 | 100 ± 0 | 100 ± 0 |
| **Mid-plane** | Axial | 99.91±0.2 | 99.9 ± 0.2 | 99.92 ± 0.1 |
|  | Sagittal | 100 ± 0 | 100 ± 0 | 100 ± 0 |
|  | Coronal | 99.83 ± 0.1 | 99.74 ± 0.2 | 99.78 ± 0.2 |
| * Development set is randomly divided into the training set and tuning set in 8:2 ratio in each 5-fold cross-validation. | | | | |

| **Supplementary Table 4. Average and standard deviation of performance measurement of contrast-enhancement classification models (%).** | | | | |
| --- | --- | --- | --- | --- |
| Body Part | Preprocessing Method | Cross-validation performances in tuning set* | | |
|  |  | Precision | Recall | Accuracy |
| Brain | Mid-plane_axial_ | 95.14 ± 2.8 | 95 ± 2.8 | 95 ± 2.7 |
| Neck |  | 98.66 ± 6.6 | 98.66 ± 6.6 | 98.66 ± 6.6 |
| Chest |  | 100 ± 0 | 100 ± 0 | 100 ± 0 |
| Abdomen |  | 100 ± 0 | 100 ± 0 | 100 ± 0 |
| Abdomen& Pelvis |  | 95.14 ± 2.8 | 95 ± 2.7 | 95 ± 2.7 |
| Brain | Mid-plane_sagittal_ | 98.15 ± 1.7 | 98 ± 1.8 | 98 ± 1.8 |
| Neck |  | 96.23 ± 4.1 | 96 ± 4.3 | 96 ± 4.3 |
| Chest |  | 100 ± 0 | 100 ± 0 | 100 ± 0 |
| Abdomen |  | 100 ± 0 | 100 ± 0 | 100 ± 0 |
| Abdomen & Pelvis |  | 99.41 ± 1.3 | 99.37 ± 1.3 | 99.37 ± 0.1 |
| Brain | Mid-plane_coronal_ | 94.5 ± 5.7 | 94.47 ± 3.9 | 94.54 ± 4.9 |
| Neck |  | 94.02 ± 3.9 | 96.66 ± 2.3 | 95.45 ± 3.2 |
| Chest |  | 100 ± 0 | 100 ± 0 | 100 ± 0 |
| Abdomen |  | 100 ± 0 | 100 ± 0 | 100 ± 0 |
| Abdomen & Pelvis |  | 100 ± 0 | 100 ± 0 | 100 ± 0 |
| * Development set is randomly divided into the training set and tuning set in a ratio of 8:2, respectively, in each 5-fold cross-validation. | | | | |

**Supplementary Figure 1. Detailed information of external validation set**


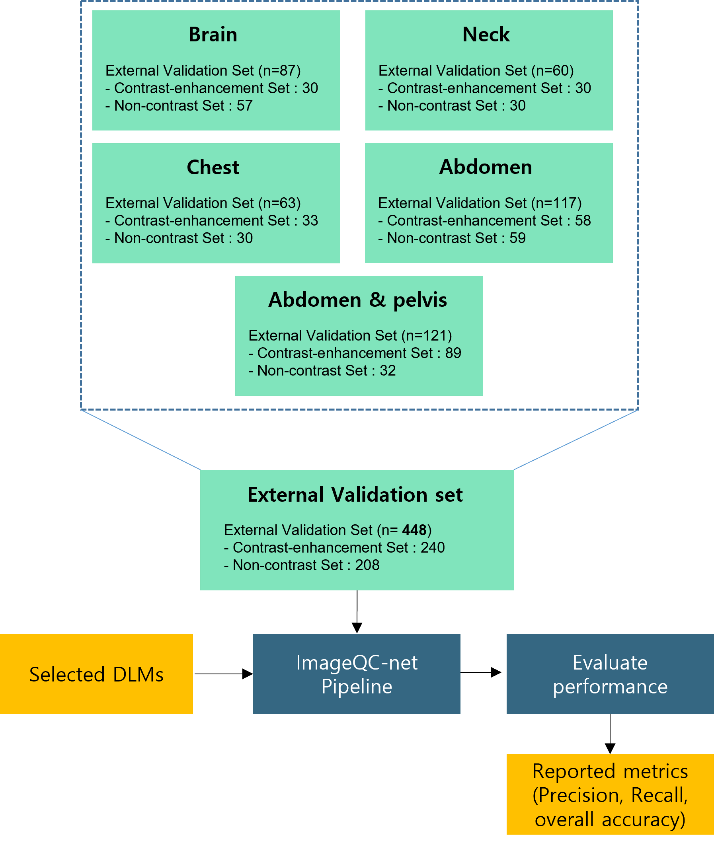


**Supplementary Figure 2. Training scheme of individual DLM
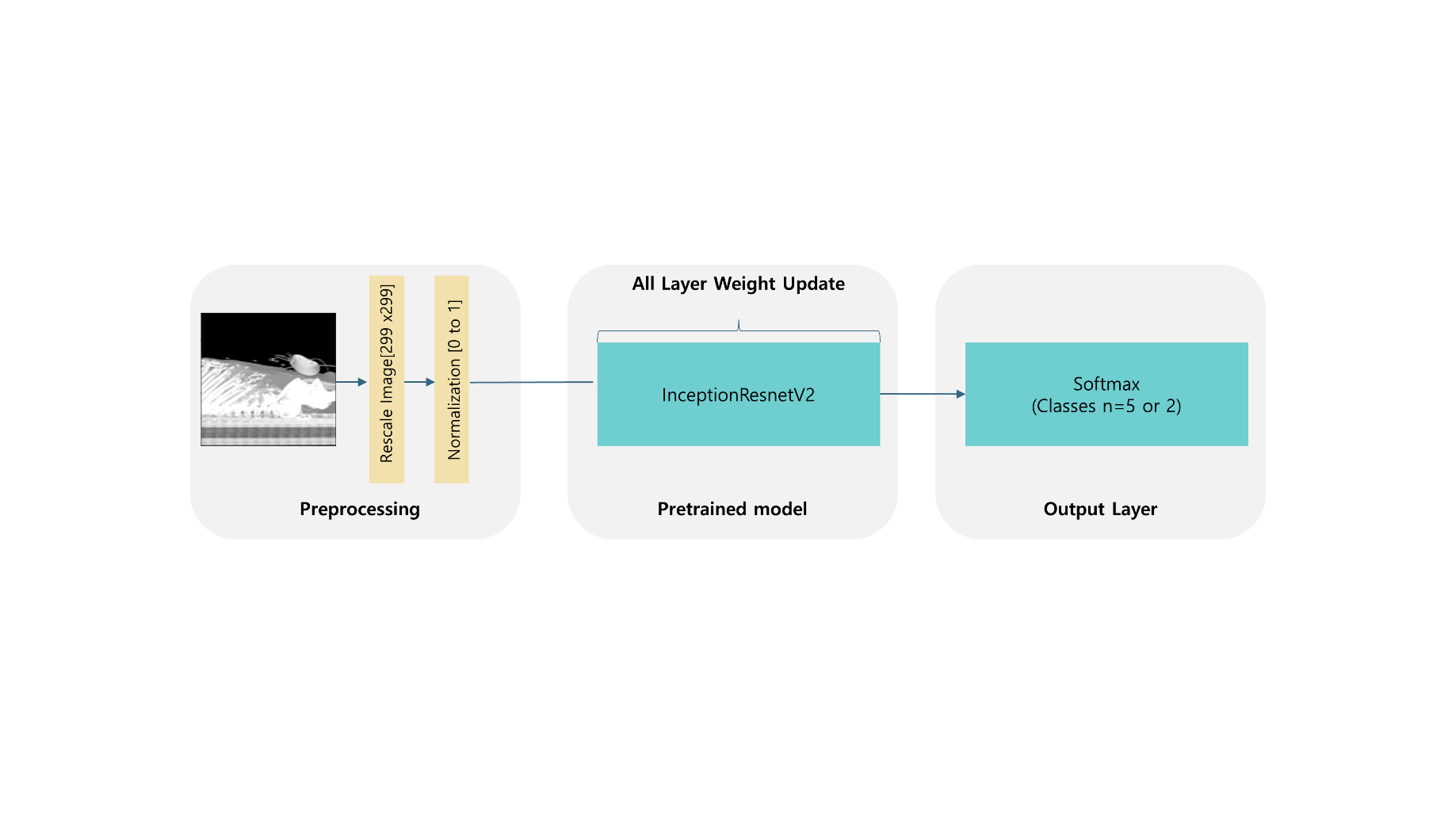
**
